# Medical Reform

**Published:** 1871-07

**Authors:** B. H. Pratt


### MEDICAL REFORM.

BY B. H. PRATT, A. M.

We are coming to it. Rather, we are going to it. Anyhow, we are approximating toward a crisis in Medicine. No; I know you don't believe it — rather, *won't* believe it — at least don't want to believe it; but you must come to it if you live long enough, my dear Doctor Oldskool. If there isn't a cloud just above the medical horison a deal bigger than a man's hand, then my optics are deceiving me most wofully. I tell you, the Practice of Medicine as laid down by the dead and gone medical lights—men whose edicts and dicta few dare question—conservators of guess-work—wallowers in the slough of doubt, and would-be wise solvers of the thousand and one conundrums which the sick bed is daily propounding—this practice cannot live. Public opinion has been slower to poke its finger into the medical, than any other pie; but now that some have been found bold enough to question even the wisdom of that grave and awe-inspiring fraternity, the Doctors, the moat that has guarded their stronghold is about to be bridged, and the outside world is bent upon passing within, and upon getting up a tremendous rattle among the dry bones there lying, and upon inspecting the interior of the whited sepulchers that have been so long deemed purity's self by reason of their external whiteness This Public Opinion, when once aroused, is a giant. The "*vox populi, vox dei*" cry is as true, as portentous, as iconoclastic, as it was in the days of old. Formidable as it is when raised in the interest of wrong, it is a perfect avalanche when its guiding star is THE RIGHT, and its swath is as wide as the round world itself. That Public Opinion—that Vox Populi—that Scourge to humbug—that Boon to philanthropy—is now marshaling its hosts—is officering its legions—is arming its braves; not hurriedly—not noisily—not boastingly—not swaggeringly—but as the fountain, insignificantly bubbles at your feet, and fed, now here, now there, by tiny rivulets, by and by grows streamlike, then silently creeps toward the mountain barrier, deepening, widening, strengthening, till ere man gives it any thought of ill, the very hills we deemed eternal rend asunder, and devastation reigns where only now dwelt sweet repose; so gather now the latent forces that are to rend the seeming adamant of medical conservatism.

When men were blind from ignorance, and only hither and yon a mind upreached above its fellows, it was not strange that superstition lent credulity—that insignia of superiority and cabalistic arts begat a reverence, nay, even awe, for its usurpers. It was no wonder that effrontery could wave its wand successfully over the gaping multitudes, when lying oracles were invested with the garb of divinity, and became the chief guide in the affairs of men. There was then that barrier betwixt the learned few and ignorant many which was deemed insurmountable, and the unfortunate ones regarded those upon the other side as especially gifted by the gods—to whom it were not only vain, but even sacrilegious to attempt to approach in mental acquirements. They allowed their credulity to be played upon as a matter of course, perceiving no trick, imagining no deceit.

That men can now affect a like superiority to their fellows, is simply preposterous. The great and glorious company of guessers is about to be forestalled. This age isn't going to submit to any such trifling with Dame Nature. She is pointing out to us her wishes, and has been doing so from creation down; but instead of endeavoring to ascertain for ourselves what those wishes are, we have been satisfied to take what some "big medicine" in days of yore has bequeathed to us; and this is medical practice pure and simple. The motto of the great bulk of our so-called medical men of to-day is, "there is nothing new under the sun." In no other department of science have we so adhered to the primitive methods as in medicine. All arts, save that of healing, are progressive. Arab like, we are satisfied to know what our fathers knew —do as our fathers did--die as our fathers died. I say we are; I mean we *seem* to be, for now that men are awakening to a new dawn of practice, a new era is imminent for which they are hoping and waiting; protesting against, but enduring the old.

It is not, in our intelligent age, to be longer credited that we must tear away so much of the human frame before we begin to rebuild it—not reasonable that the fever fire must be fanned and fed until it has well nigh consumed all that is mortal, before we apply one single effort to extinguish it—not in accordance with common sense that the patient (or victim rather) shall be denied everything that nature craves and cries out in agony for, and dosed to death's door with all the nauseous compounds of the meteria medica. We go to the books—not to the patient; we consult the Fathers—not the symptoms; the complaint, not the case, indicates a formula which is as fixed as the laws of the Medes and Persians. The practitioner opens his eyes and raises his hands in holy horror at any innovation upon his ancient thesis. We may not take a journey, stage coach fashion as of old, but accept the later and better methods of travel that enterprise has instituted. We may not mount a courier to advise with a distant friend, but speed our message on electric wings which the genius of man has discovered and tamed. But we must go through the bitter night of sickness and suffering along the old, old path, mounted on the conservative old jackass our great grandfathers strode, and raise no whimper of disobedience, forsooth; and simply because there is one science or art whose advocates and sustainers have determined shall not advance, either because they are too wrong headed or too pig headed or too lack brained to assert that advance is possible, or if so, desirable, or if so, worth the price of a reform.— Out upon such a crew! Don't they see how their villainous scheme of supporting a humbug is opening the eyes of sharp-sighted but bad men to the fact that quackery is no worse than the regular practice in its *results* ofentimes? Not that quackery and charlatanism are any the more excusable therefor, but that they are a natural result of the present system of medicine.— Charlatanism is abominable—a curse to a community, and does not meet a tithe of the scorn that should be heaped upon it. But it is the true and legitimate offspring of a mistaken and misguided parent, to wit, the medical practice of the day. And so long as the present mode of treatment obtains, so long will quacks, charlatans and the rest of the long list of medicine's parasites flourish. Revolutions are destructive —Reforms are ever beneficial. Agitation is needed and agitation is what is now going on, with results more or less gratifying, meager as they yet are. From among the long roll of honorable names which might elevate medicine into a science as exact as any of its sisterhood, are they who might achieve not distinction only, but the eternal gratitude of a world. To do this ere the combined stigma of a now widely diffused intelligence attach to a profession yearly losing ground, would claim for it the respect and plaudits of generations yet to be; while a failure to so act, and thus at last invite the usurpation of its powers by a no longer indulgent age, will bring obloquy and contempt upon it so long as it strives to hold its head above the current of popular prejudice, and go at last into unhonored oblivion.

It is not enough that men have realized the true event of "legitimate practice" and left the profession; not enough that others have wheeled about into some pathy. This is shirking the issue, and only goes to show that the honest physician *knows* how his profession hangs by a single thread. Don't desert—don't rebel— stick to your colors, but be sure they are the true ones, and then manfully battle for the Cause.

